# Imidazole-Based Ionic Liquids with BF_4_ as the Counterion Perform Outstanding Abilities in both Inhibiting Clay Swelling and Lowing Water Cluster Size

**DOI:** 10.3390/ijms22126465

**Published:** 2021-06-16

**Authors:** Qiqi Zhang, Shanmeiyu Zhang, Hui Yang, Guangling Pei, Ming Yang, Wei Zhang, Rui Chen, Jinben Wang

**Affiliations:** 1 CAS Key Lab of Colloid, Interface and Chemical Thermodynamics, Institute of Chemistry, Chinese Academy of Sciences, Beijing 100190, China; zhangqq_bift@163.com (Q.Z.); zhangsmy@iccas.ac.cn (S.Z.); yangmingchemistry@126.com (M.Y.); zhangwei2015@iccas.ac.cn (W.Z.); rui_chen1995@163.com (R.C.); jbwang@iccas.ac.cn (J.W.); 2 School of Materials Design and Engineering, Beijing Institute of Fashion Technology, Beijing 100029, China; clypgl@bift.edu.cn

**Keywords:** ionic liquids, anti-swelling, water cluster, narrow space, ultra-low permeability reservoir

## Abstract

Promoting fluid transportation in porous media has important applications in energy, pedology, bioscience, etc. For this purpose, one effective way is to prevent swelling through surface modification; however, it is far from enough in real cases, such as ultra-low permeability reservoirs and tight oils. In this study, we considered the comprehensive effects of inhibiting clay swelling, flocculation performance, reducing water clusters and interfacial tension and developed a series of imidazole-based tetrafluoroborate ionic liquids (ILs) with different lengths of alkyl chains. Through measurements of anti-swelling rates, XRD, SEM, ^17^O NMR, molecular dynamics simulation, zeta potential, flocculation evaluation, interfacial tension and a core flooding experiment based on ultra-low permeability reservoirs, the relationships between the molecular structure and physicochemical properties of ILs have been revealed. Interestingly, one of the selected ILs, imidazole-based tetrafluoroborate ILs (C_8_-OMImBF_4_), shows excellent performance, which is helpful to design an effective strategy in promoting fluid transportation in narrow spaces.

## 1. Introduction

The promotion of fluid transport in porous media has important applications in energy, soil and biological science and other fields. How to construct functional molecules to modify pore surfaces and facilitate fluid flow in narrow spaces is recognized as a worldwide problem, e.g., for ultra-low permeability reservoirs (<5 mD). Given the current contradiction between the rising energy demand and falling oil production [1], petroleum engineers pay more attention to the exploitation of unconventional oil reservoirs. A growing number of ultra-low permeability reservoirs has been proven and they account for a large proportion of the available oil in the world [2]. However, this kind of reservoirs has special reservoir characteristics and complex pore structure. Accordingly, oil development under such unconventional conditions encounters problems, such as high injection pressure, low recovery and poor economic benefits [3]. How to enhance the recovery of ultra-low permeability reservoirs has been a vital task for all the petroleum researchers.

In ultra-low permeability reservoirs, clay minerals are widely present. Clay minerals are layered silicates and these crystal platelets may have multiple octahedral or tetrahedral flakes, connected together by oxygen atoms [4,5]. Montmorillonite, which is the main component of bentonite, is composed of two layers of silica tetrahedrons with an alumina octahedron in between [6]. Moreover, Sodium montmorillonite (Na-Mt) with a cation exchange capacity (CEC) of 90 mmol/100 g, has the highest water sensitivity among many clay minerals [7]. The hydration and expansion of clay minerals cause them to disperse into fine particles with a diameter of less than 10 µm, which can easily block the mineral voids and reduce permeability. In addition, water molecules can bond closely with each other through hydrogen bonds, forming a dense hydrogen bond network. As a result, water tends to form clusters at nanoscale [8], which is another key factor of oil displacement in ultra-low permeability reservoirs. Therefore, the development of a functional molecular system that can inhibit the hydration and thickening of clay and enhance the flowability of fluid plays a vital role in the development of ultra-low permeability reservoirs.

There has been a large number of studies carried out to resolve the issue of instability posed by clay swelling. Over the past decades, numerous chemicals have been used as clay inhibitors, including inorganic salts, silicates, polymers, organic amines, ammonium compounds and so on. Recently, as one of the new materials as promising clay inhibitors, ionic liquids (ILs) have been widely investigated. ILs usually refer to an organic salt with a melting point below 100 °C, which is a “green chemical substance” [9]. Luo et al. [10] revealed that 1-octyl-3-methylimidazole tetrafluoroborate has better shale inhibition than potassium chloride. Yang et al. [11] also studied 1-vinyl-3-ethylimidazole bromide monomer and its corresponding homopolymers as clay inhibitors, both of which show good inhibition properties. The type of ionic liquids cation or anion groups affects the size, solubility, melting temperature and hydrophilicity/hydrophobicity of clay and correspondingly affects the anti-swelling performance of clay. Yang et al. [12] evaluated the effect of cationic components on the inhibitory performance of ionic liquids and discussed how the alkyl chain length of vinyl imidazolium ILs affects their inhibitory effect. Experimental results found that with the shortest chain of ethyl, ILs have the best ability to inhibit hydration. Moreover, as the alkyl chain length increases, the inhibition performance of ILs decreases. Khan et al. [13] studied four different ILs with the same cationic group 1-allyl-3methylimidazole but different anions (bromide, iodide, chloride and dicyanamide) and studied their influence on clay inhibition. The results were that the ILs with different anions reduce the clay swelling and the clay swelling does not strongly depend on the type of anion in imidazolium-based ILs. Xu et al. [14] found that the ionic liquid 1-octyl-3-methylimidazolium bromide has good shale hydration inhibition properties and it could effectively reduce surface tension even at low concentrations. Jia et al. [15] studied the inhibitory ability of 1-hexyl-3-methylimidazole bromide (BMH) and 1,2-bis(3-hexylimidazolium-1-yl) ethane bromide (HMH). They demonstrated both BMH and HMH effectively inhibit the shale hydration and swelling. HMH, as a gemini surface active ionic liquid, compared with BMH, was good in entering the interlayer space of clay, lowering the zeta potential, forming a hydrophobic barrier layer and lowering surface tension, which was the reason for its excellent inhibition performance. On this basis, our group devotes to exploring the potential of imidazole-based tetrafluoroborate ILs in improving oil recovery in ultra-low permeability reservoirs, considering not only the nature of the minerals but also the size of fluid aggregates and the interfacial activity.

Therefore, this paper mainly considers the combined effects of imidazole-based tetrafluoroborate ILs with different lengths of alkyl chains on inhibiting sodium bentonite (Na-bent) swelling, reducing water clusters, flocculation performance and lowering interfacial tension. Different test methods, including ^17^O NMR, molecular dynamics simulation, anti-swelling rate, XRD, SEM, zeta potential, flocculation experiment and interfacial tension, were conducted. In order to expand the application of ILs in improving oil recovery in ultra-low permeability reservoirs, core flooding experiments were carried out. The results showed that the imidazole-based tetrafluoroborate ILs (C_8_-OMImBF_4_) is an excellent candidate for the oilfield practice due to its high performance.

## 2. Results and Discussion

**Figure 1:**
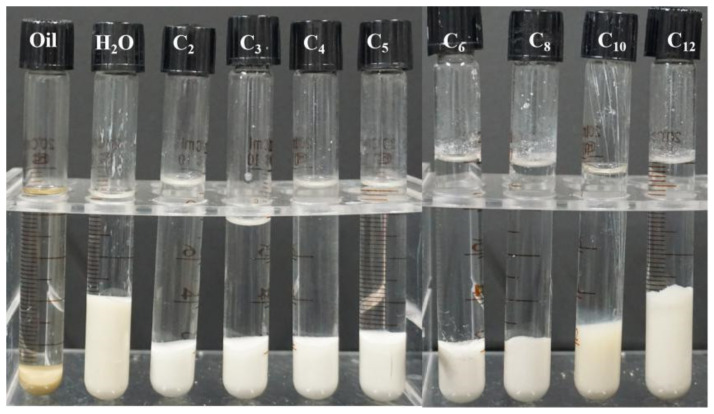
Anti-swelling performance of ILs (C_2_-EMImBF_4_–C_12_-MImBF_4_) at high concentrations.

It can be seen from Figure 1 that ILs with different alkyl chain lengths have a positive anti-swelling effect on Na-bent at high concentration. After centrifugation, the upper liquid of ILs at high dosage is relatively clear. The anti-swelling rates of ILs are shown in Table 1 and the decreasing order is: (C_2_-EMImBF_4_, C_3_-PMImBF_4_, C_4_-BMImBF_4_) > (C_5_-PnImBF_4_, C_6_-HMImBF_4_, C_8_-OMImBF_4_) > C_10_-DMImBF_4_ > C_12_-MImBF_4_.

When Na-bent is immersed in water, due to the action of hydration and electrostatic repulsion, the interlayer spacing of Na-bent is enlarged microscopically and the particles repel each other, so that the mixture of water and Na-bent form a stable suspension. When ILs with a high positive charge density are added, they insert between the layers of Na-bent particles to supplement the positive charge, weakening the electrostatic repulsion between Na-bent layers and reducing the layer spacing, which results in a reduction in the volume of Na-bent on a macroscopic scale. The short alkyl chain is conducive to the solid–liquid separation, further explained by the following discussion.

**Figure 2:**
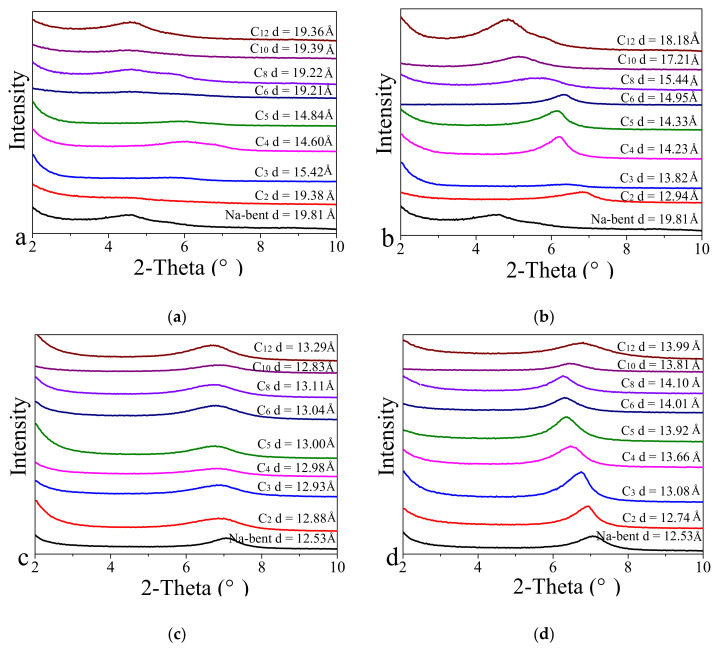
XRD spectra of wet and dry Na-bent samples in the presence of different ILs: (**a**) wet sample at low concentration; (**b**) wet sample at high concentration; (**c**) dry sample at low concentration; (**d**) dry sample at high concentration.

**Figure A1:**
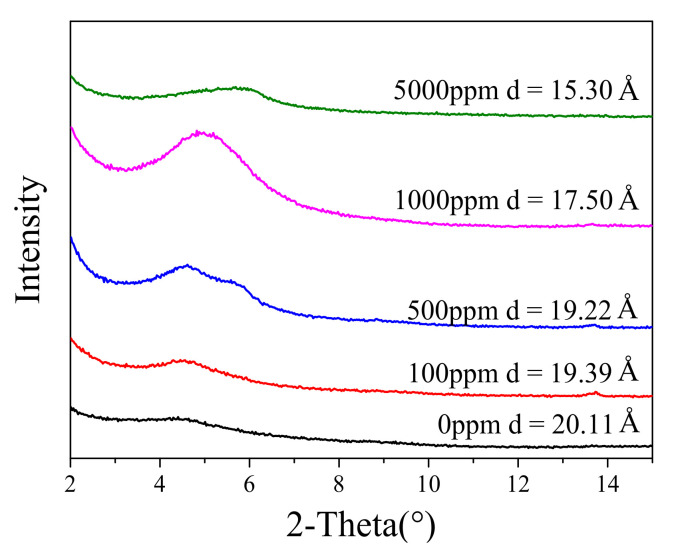
The d-spacing of wet Na-bent under the action of C_8_-OMImBF_4_ at different concentrations.

In order to explain the role of ILs in the hydration and swelling processes of Na-bent, the interlayer spacing (d-spacing) of Na-bent was measured by XRD. The original d-spacing of Na-bent is 12.53 Å and the d-spacing of the fully hydrated clay particles is 19.81 Å. The d-spacing significantly increases due to hydration expansion. After the addition of ILs, the interlayer spacing of hydrated Na-bent is compressed, as shown in Figure 2a,b, especially for high ILs dosage. The cationic groups of ILs can be adsorbed on the surface of Na-bent through electrostatic interactions and balance the charge distribution, thereby reducing hydration repulsion and interlayer spacing. It can be seen that the d-spacing almost increases with the increase of the alkyl chain length of ILs, because of the size effect. Moreover, at the concentration of 5000 ppm, the d-spacing of Na-bent decreases significantly under the action of C_8_-OMImBF_4_, compared with lower concentrations, as shown in Figure A1.

**Figure A2:**
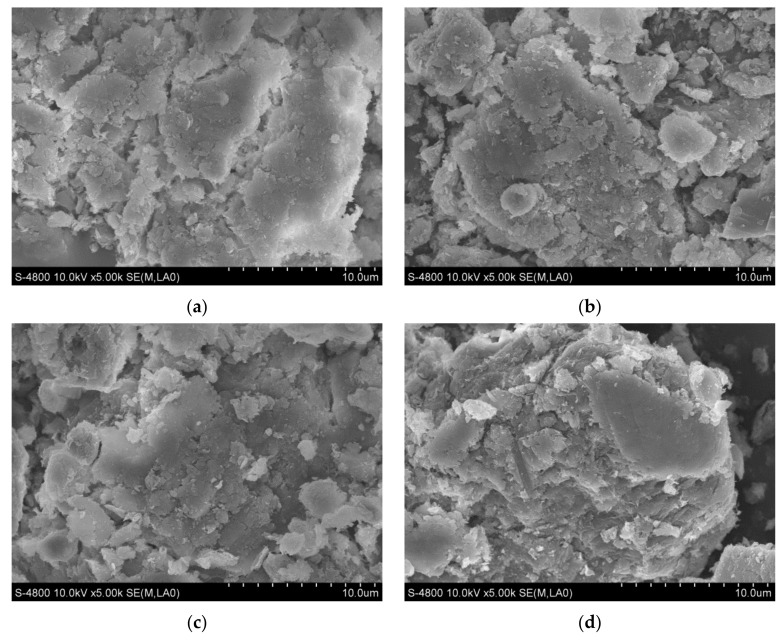

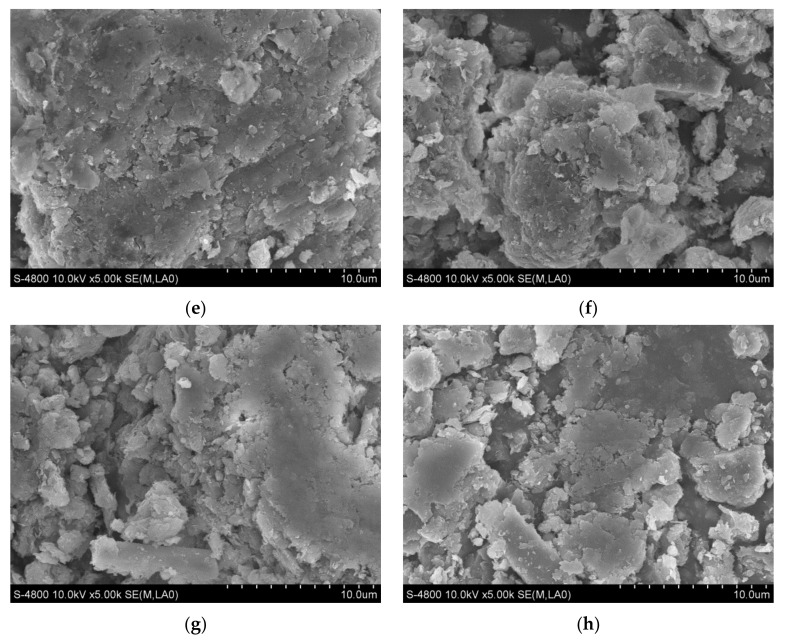
The SEM of original Na-bent and Na-bent treated by H_2_O and ILs: (**a**) original Na-bent; (**b**) hydrated Na-bent; (**c**) C_2_-EMImBF_4_; (**d**) C_3_-PMImBF_4_; (**e**) C_4_-BMImBF_4_; (**f**) C_5_-PnImBF_4_; (**g**) C_6_-HMImBF_4_; (**h**) C_8_-OMImBF_4_.

The interlayer spacing of dry Na-bent samples with different concentrations of ILs is shown in Figure 2c,d, which can quantitatively describe the degree of intercalation of ILs in the middle layer of Na-bent. The interlayer spacing of Na-bent increases to 12.88–13.29 Å at low concentration and increases a little more at high concentration, indicating that the ILs are successfully inserted into the interlayer space. The results are consistent with a previous report [15]. Moreover, the surface morphology of sodium bentonite after treatment with deionized water and ILs was analyzed by SEM, as shown in Figure A2. The original surface of bentonite was dense and smooth. The sodium bentonite contacted with deionized water became swollen, disintegrated and then dispersed. After adding ILs, the surface dispersion of sodium bentonite was reduced.

**Figure 3:**
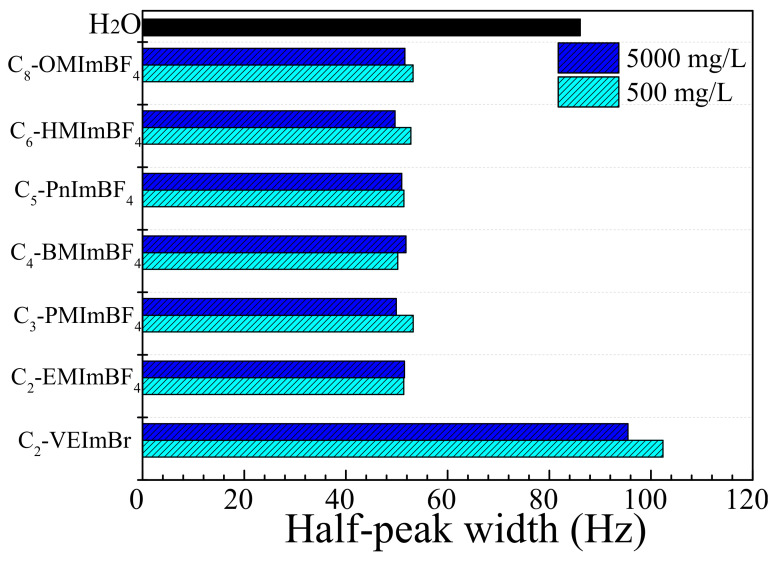
Half-peak width of the ^17^O NMR spectra of H_2_O and ILs at different concentrations.

The half-width of ^17^O NMR relates to the average relative size of the liquid water cluster structure. The wider the spectrum, the larger the cluster; the narrower the spectrum, the smaller the cluster [16]. Figure 3 shows the half-peak width of the ^17^O NMR spectra of H_2_O and ILs (C_2_-EMImBF_4_–C_8_-OMImBF_4_) at different concentrations. Due to the poor solubility of C_10_-DMImBF_4_ and C_12_-MImBF_4_ in water at room temperature, their oxygen spectra are not applied. By comparison, the half-width of each selected IL at both low and high concentrations is lower than that of pure water (peak width = 86.1 Hz), indicating that ILs have the ability to reduce water clusters. As the concentration increases, there is a tendency for the half-peak width to decrease, which might be due to the increase in the concentration of BF_4_^−^ and the increased polar effect of F^−^, resulting in the formation of stronger hydrogen bonds with water than those between water molecules. There is little change in the measured half-value width of ILs with different alkyl chains at the same concentration, indicating that the length of the alkyl chain has little effect on reducing water clusters. For example, the half-widths of C_3_-PMImBF_4_, C_5_-PnImBF_4_ and C_6_-HMImBF_4_ are 49.9 Hz, 51.0 Hz, 49.7 Hz, respectively, which are a bit better than C_2_-EMImBF_4_ (51.6 Hz), C_4_-BMImBF_4_ (51.8 Hz) and C_8_-OMImBF_4_ (51.6 Hz).

**Figure A3:**
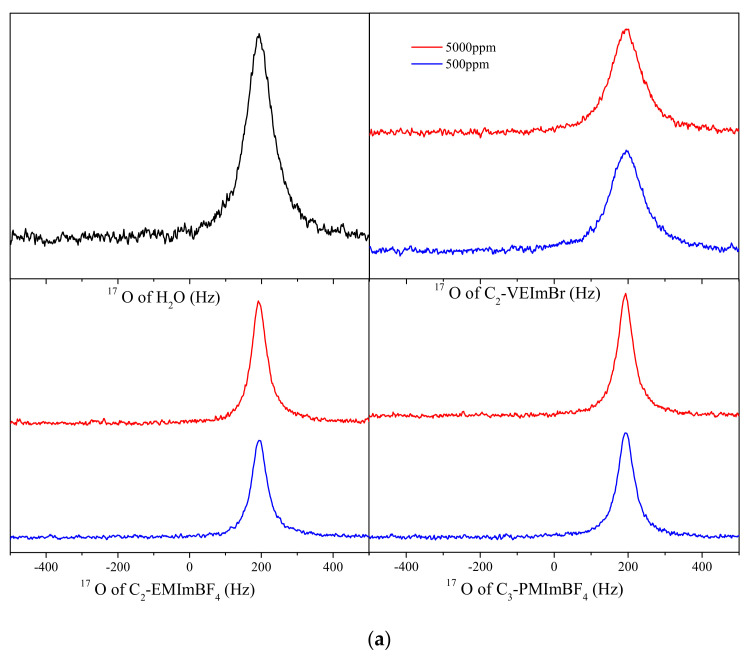

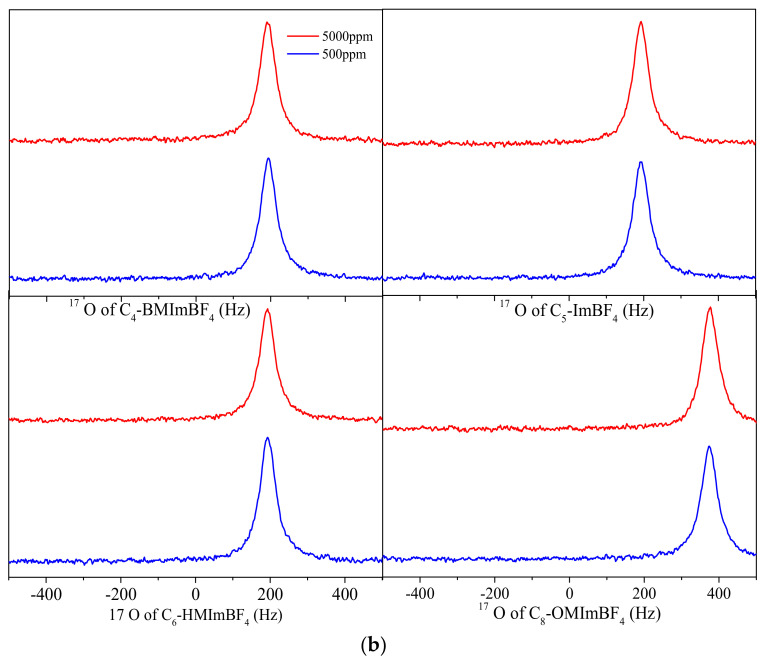
The ^17^O NMR spectra of H_2_O, C_2_-EMImBF4–C_8_-OMImBF_4_ and C_2_-VEImBr at concentrations of 500 ppm and 5000 ppm, respectively.

The ionic liquids of C_2_-VEImBr are reported to have excellent ability to inhibit hydration in the past study [11]. In comparison, we also tested the ^17^O-NMR half-peak width of C_2_-VEImBr and they are 102 Hz and 95.5 Hz at low and high concentrations, respectively. The values are much bigger than that of the tetrafluoroborate ILs series, indicating the weak ability of C_2_-VEImBr in reducing water molecular clusters. The ^17^O NMR spectra of H_2_O, C_2_-EMImBF_4_–C_8_-OMImBF_4_ and C_2_-VEImBr are also shown in Figure A3.

**Figure 4:**
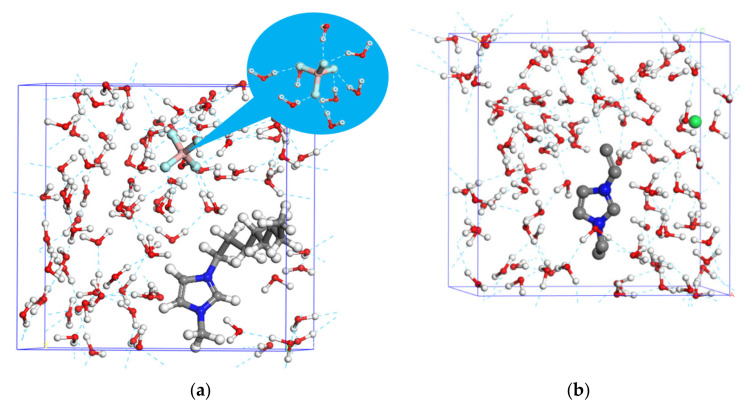
Interaction between different kinds of ILs and water molecules:(**a**) C_8_-OMImBF_4_; (**b**) C_2_-VEImBr).

To further reveal the interaction mechanism between ILs and water molecules, the kinetic energy was calculated through MD simulation, as shown in Figure 4. It is concluded that several hydrogen bonds form between the BF_4_^−^ group and water molecules (see inset Figure 4), resulting in disassembling big water clusters into small ones. In comparison, the C_2_-VEImBr molecule hardly forms hydrogen bonds with water molecules, mainly because of the good hydration cooperation between C_2_-VEImBr and water molecules and, therefore, the network structure of water cluster cannot be destroyed. The results support the conclusion from ^17^O NMR results.

**Figure 5:**
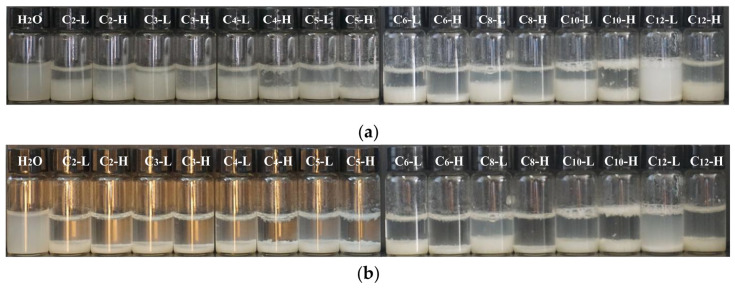
Flocculation performance of ILs at low (L, low concentration) and high (H, high concentration) concentrations after being added for: (**a**) 1 min; (**b**) 30 min.

As shown in Figure 5a, when ILs are added to the Na-bent dispersion system, obvious flocculent precipitation is observed compared with pure water. After 30 min, as shown in Figure 5b, the suspension is clearer, especially for C_2_-EMImBF_4_–C_8_-OMImBF_4_ systems at high concentration. The flocculation performance of C_8_-OMImBF_4_ at high concentration is the best in our study. The reason for the rapid flocculation of ILs comes from the high positive charge density and hydrophobicity.

**Figure A4:**
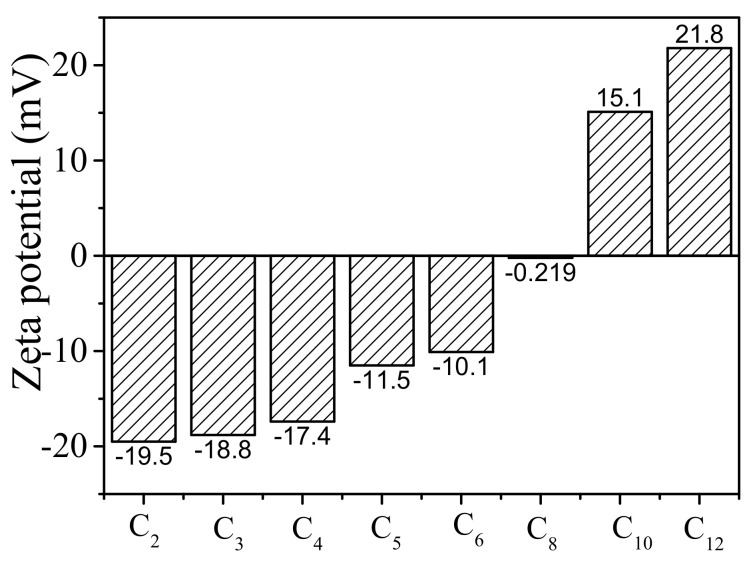
Zeta potential of ILs with different alkyl chains at high concentration.

**Figure A5:**
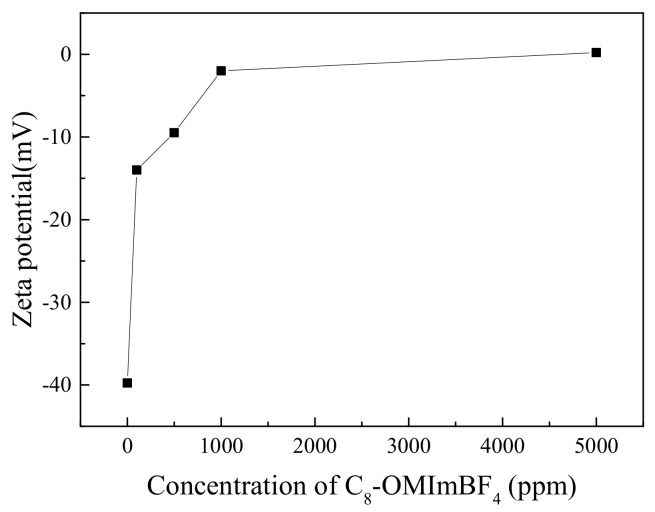
Zeta potential of C_8_-OMImBF_4_ at different concentrations.

The surface of Na-bent particles is negatively charged due to isostructural substitution, which means that cations are required to achieve charge balance. When the ILs molecules are adsorbed onto the surface of solid particles, the negative charge on the surface of bentonite particles is neutralized and the electrostatic repulsion force is reduced. It is reported in the literature that an absolute value of zeta potential higher than 30 mV will ensure the stability of the colloidal system [17]. The zeta potential of Na-bent suspension in deionized water is -33.6 mV, showing good stability. The zeta potential of ILs with different alkyl chains at a high concentration are shown in Figure A4, which shows that as the alkyl chain length increases the ability of ILs to inhibit the double electron layer of the Na-bent suspension increases, except for C_10_-DMImBF_4_ and C_12_-MImBF_4_. The zeta potential almost reaches the isoelectric point (IEP) at high concentrations of C_8_-OMImBF_4_. As shown in Figure A5, the zeta potential is lower than the isoelectric point when the concentration is lower than the high concentration of C_8_-OMImBF_4_. At the isoelectric point (IEP), a relatively fast sedimentation behavior can be observed. On the one hand, after imidazole cations are adsorbed on the surface of particles due to the action of charge, the hydrophobic effect of the extended hydrophobic tail chain is conducive to solid–liquid separation. On the other hand, when ILs with high charge density interact with colloid particles with opposite charge, there will be charge patch effect, so charge patch is also one of the additional gravitational effects. The heterogeneous particle surfaces caused by charge patches generate electrostatic attraction to each other, which intensifies the solid–liquid separation process. In addition, C_10_-DMImBF_4_ and C_12_-MImBF_4_ reverse the zeta potential to +15.1 mV and +21.8 mV, respectively. This might be because the IL with positive charge is adsorbed on the surface of bentonite particles and the charge is neutralized, making the potential from negative to positive.

The interfacial tensions of C_6_-HMImBF_4_, C_8_-OMImBF_4_, C_10_-DMImBF_4_, C_12_-MImBF_4_ ILs with oil were 14.40, 9.38, 5.40, 0.61 mN/m, respectively. This addition of ILs significantly reduced the interfacial tension of the oil–water interface, which was beneficial to emulsify the crude oil into an emulsion, aggregate and form an oil zone and change the fluidity of the crude oil.

**Figure 6:**
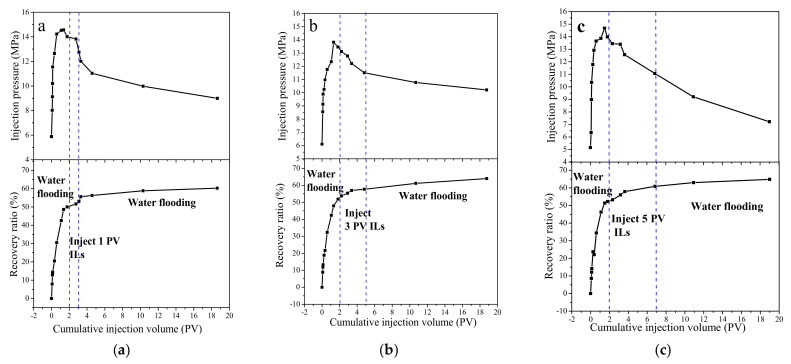
Injection volume curves and recovery ratio curves during oil displacement: (**a**) 1 PV; (**b**) 3 PV; (**c**) 5 PV of C_8_−OMImBF_4_ ILs injection.

Combining the abilities of decreasing water cluster size, reducing clay swelling and enhancing flocculation, C_8_-OMImBF_4_ with higher oil–water interfacial activity was selected to perform the oil displacement experiment in the condition of an ultra-low permeability core. The composition and salinity of Xing Hebei Chang-6 simulate formation water is shown in Appendix A, Table A1. The parameters of the three cores used in the experiment are shown in Table A2. The results of injection pressure and recovery ratio are shown in Figure 6. During the displacement process, the injection pressure first increased, and reached the maximum pressure, and then decreased. Meanwhile, the recovery ratio was recorded until no oil was produced. The enhanced oil recovery is 10.2%, 12.1%, 12.6%, respectively, after injecting 1, 3, 5 PV C_8_-OMImBF_4_ agents.

C_8_-OMImBF_4_ with imidazole cation structure is easily adsorbed onto clay through electrostatic interaction, which can compensate the negative sites of Na-bent and compress the electrical double layer. Moreover, ILs can insert into the interlayer space of Na-bent and expel some interlayer water molecules. Hence, the trend of Na-bent in water adsorbing can be reduced. At the same time, ILs are capable of leading the Na-bent to be more hydrophobic, thereby a hydrophobic barrier that restricts water intrusion forms. Because of the special structure and properties of C_8_-OMImBF_4_, they effectively reduce the clusters of water molecules and favor the fluidity through tiny channels. Moreover, C_8_-OMImBF_4_ significantly reduces the interfacial tension of the oil–water interface (Figure A3), which is beneficial when interacting with crude oil and forming an oil disperse system. Altogether, ILs with distinguishing molecular structure and characteristics will help to develop a new guide for practical applications.

## 3. Materials and Methods

### 3.1. Materials

**Figure 7:**
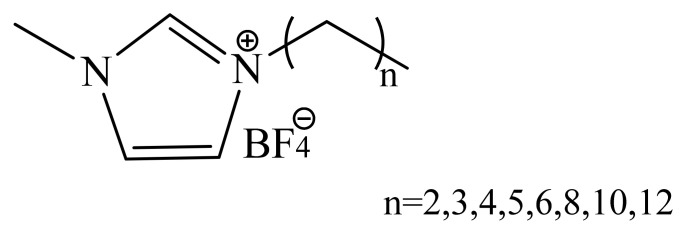
Structure formula of imidazole-based ILs with different lengths of alkyl chain.

1-ethyl-3-methylimidazole tetrafluoroborate (C_2_-EMImBF_4_), 1-propyl-3-methylimi-dazole tetrafluoroborate (C_3_-PMImBF_4_), 1-butyl-3-methylimidazole tetrafluoroborate (C_4_-BMImBF_4_), 1-pentyl-3-methylimidazole tetrafluoroborate (C_5_-PnMImBF_4_), 1-hexyl -3-methylimidazole tetrafluoroborate (C_6_-HMImBF_4_), 1-octyl-3-methylimidazole tetrafluoroborate (C_8_-OMImBF_4_), 1-decyl-3 methylimidazole tetrafluoroborate (C_10_-DMImBF_4_) and 1-dodecyl-3-methylimidazole tetrafluoroborate (C_12_-MImBF_4_), 1-vinyl-3-ethylimidazole bromide (C_2_-VEImBr) were supplied by the Lanzhou Institute of Chemical Physics, Chinese Academy of Sciences, China. Their purity is 99%. The structure formula of imidazole-based ionic liquids (ILs) is presented in Figure 7. Sodium bentonite (Na-bent) was purchased from the Beijing Bailing Wei Technology Co., Ltd. (China). Deionize water (DW) was used to prepare the solutions. The crude oil from the XingHebeiChang-6 oilfield (China) was used for core displacement experiments. The ultra-low permeability cores were purchased from Jiangsu Lian you Scientific Research Instrument Co., Ltd.

### 3.2. Experimental Methods

#### 3.2.1. Anti-Swelling Performance

Centrifugation. The performance evaluation method of the anti-swelling agent refers to the centrifugal method specified in the People’s Republic of China Petroleum and Natural Gas Industry Standard SY/T5971-94 “Evaluation of the Performance of Clay Stabilizers for Water Injection in Oilfields”. First, 0.50 g of Na-bent powder was put into a 10 mL centrifuge tube, with 10 mL of anti-swelling agent solutions added. Then tubes were shaken well and stored at room temperature for 2 h. Finally, they were centrifuged at 1500 r/min for 15 min, with the volume of the expanded Na-bent as *V*1. The anti-swelling agent solution was replaced with 10 mL of distilled water and kerosene and the swelling volumes of Na-bent in water and kerosene were determined as *V*_2_ and *V*_0_, respectively.

The anti-swelling rate formula is as follows:

(1) $$B_{1}=\frac{V_{2}-V_{1}}{V_{2}-V_{0}}\times 100\%,$$

where *B*_1_ is the anti-swelling rate, %; *V*_0_ is the swelling volume of Na-bent in kerosene, mL; V1 is the swelling volume of Na-bent in the clay stabilizer solution, mL; *V*_2_ is the swelling volume of Na-bent in water, mL. In our measurements, *V*_0_ = 1.0 mL and *V*_2_ = 4.5 mL.

#### 3.2.2. Investigate the Influence of ILs on the Clusters of Liquid Water Molecules

^17^O NMR. The ^17^O nuclear magnetic resonance (^17^O NMR) was used to study the effect of ionic liquids on the clusters of liquid water molecules. All ^17^O NMR chemical shifts and half widths were conducted on a Bruker AV Ⅱ 400 WB superconductor spectrometer and obtained at 67.786 MHz using “z g” pulse. Each free induction decay (FID) had 256 scans with a recycle delay of 0.2 s. In this experiment, ^17^O chemical shifts and half-widths were expressed in Hz (not in the usual ppm), because ultra-low concentrations result in low chemical shifts. The reproducibility of the chemical shifts was better than 2 Hz.

MD Simulations. Molecular dynamics simulations with different modules were computed by the Materials Studio 7.0 software. The simulation proceeded as follows: (i) Box 1, with 100 water molecules, was constructed by the Amorphous cell tool and Box 2, with the anion BF4^−^, cation group 1-octyl-3-methylimidazolium of C_8_-OMImBF_4_ and 100 water molecules, was constructed by the Amorphous cell tool. Box 3, with the anion Br^−^, cation group 1-vinyl-3-ethylimidazolium of C_2_-VEImBr and 100 water molecules, was constructed by the Amorphous cell tool. (ii) The geometry optimization task on the three boxes was performed in the Forcite module. (iii) Dynamic simulation on Box 2 and Box 3 was performed at 298.15 K in the Forcite module. By using the amorphous cell tool, a solution box of 7.77 × 7.77 × 7.77 Å (α = β = γ = 90°) was created including one model molecule and 100 water molecules whose density was set to 1.00 g/cm^3^. During the entire simulation process, charges were analyzed using the Qeq method, the ensemble chosen was NPT and Dreiding was selected for the force field. The average temperature accurately reached the set, so we choose to discuss the kinetic energy in the average state.

#### 3.2.3. Preparation of ILs/Na-Bent Composites

First, ILs of different alkyl chains were prepared at 25 °C. The mass concentrations were 500 and 5000 ppm for C_2_-EMImBF_4_– C_12_-MImBF_4_ and C_2_-VEImBr, respectively. Then, Na-bent (14 g) was suspended in 350 mL of deionized water and the mixture was stirred vigorously for 24 h for complete hydration. Then, ILs (4 mL) was added to 4 mL of freshly prepared supernatant Na-bent suspensions. The Na-bent/ILs mixture suspensions were obtained after stirring for another 24 h. After stirring, centrifugal treatment was taken at 8000 r/min for 20 min and the mixture was cleaned several times to remove the unreacted ILs. After centrifugation, the sediment of modified Na-bent was collected and divided into two parts. One part was used to measure the XRD of the wet sample and the other precipitate was dried at 105 °C and grinded into fine powder for characterization tests.

#### 3.2.4. Characterization Methods

XRD. XRD can be used to characterize and determine changes in the spacing between layers of Na-bent. The wet precipitates and dried powder were measured directly by a wide-angle X-ray diffractometer (Empyrean, Netherlands). XRD analysis was performed by an Empyrean X-ray generator with Cu Kα radiation, λ = 1.5406 Å, at a voltage of 40 kv and a current of 40 mA. The basal spacing (d-spacing) was analyzed using Bragg equation. The scattering angle (2θ) range of the sample test is 2°–10°.

Zeta Potential. Zeta potential was determined by a Nano Brook Omni potential analyzer (Zetasizer Nano ZS ZEN3600, America) and the test temperature was 25.0 ± 0.1 °C. To determine the zeta potential of the supernatant of the Na-bent dispersion system in the presence of no ILs (Na-bent suspensions) and ILs, each experiment was repeated three times and the average value was taken.

SEM. The surface morphologies of the samples of raw Na-bent, hydrated Na-bent and Na-bent modified with different ILs were observed with a scanning electron microscope (SEM). Power samples were attached on the top of an aluminum stopper by means of carbon conductive adhesive tape and spray metal for 2 min.

Flocculation Experiment. A total of 30 mg of Na-bent powder was put into the 10 mL bottle and 5 mL of ILs were added. After performing an ultrasound for 30 min (KQ3200E, Kunshan Instrument Company, China), the flocculation phenomenon was recorded by taking photos after 1 min and 30 min.

Interfacial Tension. The instrument used was a TX550C full-scale interfacial tension tester produced by Biao Wei Industries (US). The temperature was set at 55 °C. This experiment used the rotating droplet method to measure the interfacial tension of two miscible fluids.

#### 3.2.5. Core Flooding Experiment

**Figure 8:**
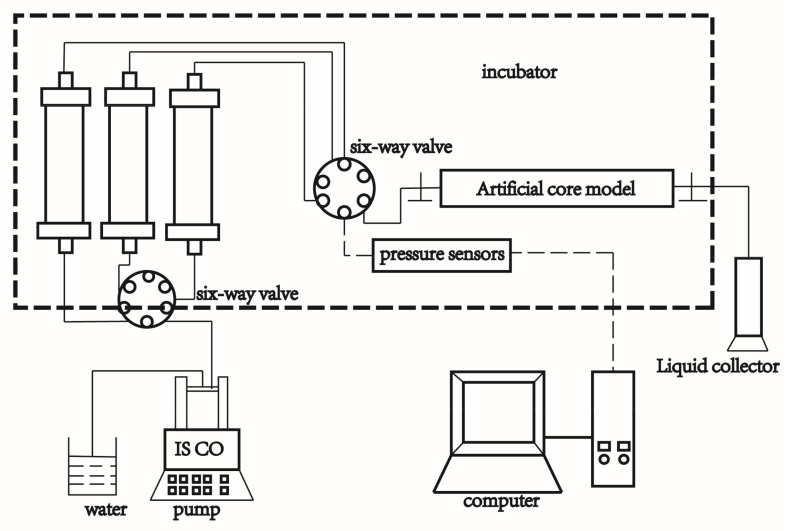
Diagram of the core displacement experiment.

The oil displacement experiment diagram is shown in Figure 8. Rubber sleeve liners were present in the core holder. The ultra-low permeability core was inserted into the rubber sleeve and placed in the core holder. The experimental procedure was conducted following four steps:

1. Simulate formation water in the ultra-low permeability core using a vacuum pump until the bubble production is negligible from the outlet;
2. Saturate crude oil in the above core until the water cut produced from the outlet is <1%;
3. Simulate formation water flooding until the effluent water cut is >98%;
4. Inject 1, 3 and 5 PV of C_8_-OMImBF_4_ solution into the cores, respectively; simulate formation water flooding again until the effluent water cut is >98% again.

A seal test was carried out by injecting water in the annulus of the core holder with the maintained overburden pressure at 2–3 MPa from the top valve of the core holder. The IOR experiments were performed in ultra-low permeability cores, injecting C_8_-OMImBF_4_ at a concentration of 800 ppm. The core size was Φ2.5cm × 8 cm (diameter × length). Moreover, the injection rate was 0.025 mL/min. The pressure data, oil production data and water production data were all recorded to analyze the recovery ratio and water cut over the injected pore volume.

## 4. Conclusions

In this work, we studied the properties, including inhibiting clay swelling, lowing water cluster size, flocculation property and interfacial performance, of imidazole-based tetrafluoroborate ILs with different lengths of alkyl chains. Through the anti-swelling rate test, XRD, SEM, ^17^O NMR, zeta potential, flocculation experiment and interfacial tension test, ILs, such as C_2_-EMImBF_4_, C_3_-PMImBF_4_, C_4_-BMImBF_4_, C_5_-PnImBF_4_, C_6_-HMImBF_4_ and C_8_-OMImBF_4_, can effectively inhibit swelling performance of clay and reduce the effect of water molecule clusters. The addition of tetrafluoroborate ionic liquids can reduce the half-peak width of pure water by up to 40%, in our study, indicating the reduction of the phenomenon of water molecular clustering, because of the interactions between BF_4_^−^ groups and water molecules. As the zeta potential of C_8_-OMImBF_4_ almost reaches the isoelectric point (IEP), it has an excellent flocculation effect compared to other selected ILs. With suitable lengths of alkyl chain, ILs are also easy to adsorb onto clay through electrostatic interactions and insert into the interlayer space of Na-bent, forming a hydrophobic barrier that restricts water intrusion and expels interlayer water molecules. The results not only provide a micro explanation for inhibiting clay swelling and lowering water cluster size, but also recommended a general strategy for applications in narrow spaces.

## Figures and Tables

**Table 1:** Anti-swelling rate (B_1_) of ILs at high concentration.

	C_2_-EMImBF_4_	C_3_-PMImBF_4_	C_4_-BMImBF_4_	C_5_-PnImBF_4_	C_6_-HMImBF_4_	C_8_-OMImBF_4_	C_10_-DMImBF_4_	C_12_-MImBF_4_
V_1_/mL	2.20	2.40	2.20	3.00	3.20	3.00	3.60	4.20
B_1_/%	65.7	60.0	65.7	42.9	37.1	42.9	25.7	8.60

## Data Availability

Not applicable.

## Appendix A

**Table A1:** Composition and salinity of XingHebeiChang-6 simulate formation water.

Type	pH	HCO_3_^−^	Cl^−^	SO_4_^2−^	Ca^2+^	Mg^2+^	K^+^+Na^+^	Salinity	Water Type
Xing Hebei Chang-6	5.8	125	49071	265	15407	302	13758	81910	CaCl_2_

**Table A2:** Parameters of the three cores.

Core Number	Air Permeability/mD	Porosity/%	Oil Saturation/%
1	0.989	11.7	93.5
2	0.994	12.0	91.5
3	1.014	12.3	92.6
